# Prognostic Value of Metabolic–Muscle Reserve Index in HR+/HER2− Metastatic Breast Cancer Treated with CDK4/6 Inhibitors

**DOI:** 10.3390/jcm15218641

**Published:** 2026-11-06

**Authors:** Nagihan Kolkıran, Atike Pınar Erdoğan, Mustafa Şahbazlar, Gözde Mütevelizade, Ferhat Ekinci

**Affiliations:** 1 Department of Medical Oncology, Manisa Celal Bayar University Faculty of Medicine, Manisa 45030, Turkey; nagihan.kolkiran@gmail.com (N.K.); m_sahbazlar@hotmail.com (M.Ş.); drferhatekinci@hotmail.com (F.E.); 2 Department of Nuclear Medicine, Manisa Celal Bayar University Faculty of Medicine, Manisa 45030, Turkey; gozdemutevelizadee@gmail.com

**Keywords:** metastatic breast cancer, CDK4/6 inhibitors, overall survival, progression-free survival

## Abstract

**Background/Objectives:** Despite the established benefits of cyclin-dependent kinase 4/6 (CDK4/6) inhibitors, survival outcomes remain heterogeneous among patients with hormone receptor-positive (HR+)/human epidermal growth factor receptor 2-negative (HER2−) metastatic breast cancer. We developed the Metabolic–Muscle Reserve Index (MMRI), integrating tumor metabolic activity, biochemical status, and skeletal muscle quantity, and investigated its relationship with progression-free survival (PFS) and overall survival (OS). **Methods:** We conducted a retrospective study involving 168 patients with HR+/HER2− metastatic breast cancer who were treated with CDK4/6 inhibitors. MMRI incorporated baseline PET/CT-derived maximum standardized uptake value (SUVmax), serum lactate dehydrogenase (LDH), albumin, and CT-derived psoas volume index (PVI). Receiver operating characteristic (ROC) analysis identified an exploratory MMRI cut-off. We examined survival using Kaplan–Meier analysis and Cox regression. **Results:** ROC analysis identified an MMRI cut-off of 10.68 for mortality discrimination, with an AUC of 0.642 (95% CI: 0.563–0.716). Patients with MMRI > 10.68 had shorter median PFS (14.1 vs. 28.1 months; *p* < 0.001) and OS (29.7 months vs. not reached; *p* < 0.001) than those with MMRI ≤ 10.68. In multivariable Cox analysis, higher MMRI was associated with shorter PFS (HR: 1.857, 95% CI: 1.329–2.596; *p* = 0.0003) and OS (HR: 1.974, 95% CI: 1.368–2.846; *p* = 0.0003). **Conclusions:** Patients with higher MMRI values experienced shorter PFS and OS during CDK4/6 inhibitor treatment for HR+/HER2− metastatic breast cancer. Given its modest discriminative performance, these findings remain exploratory and require prospective external validation before clinical application.

## 1. Introduction

Breast cancer leads global cancer diagnoses and remains a major cause of death among women [1]. HR+/HER2− disease constitutes the predominant molecular subtype in metastatic breast cancer (MBC) and represents the largest therapeutic subgroup in advanced settings [2]. The addition of CDK4/6 inhibitors to endocrine therapy has improved outcomes in HR+/HER2− metastatic breast cancer, with long-term PALOMA, MONALEESA, and MONARCH trial results demonstrating benefits in survival [3,4,5,6]. Recent updates from MONALEESA studies have confirmed durable overall survival benefit across clinically relevant subgroups, establishing CDK4/6 inhibition as the backbone of first-line therapy [4,5,6].

Although CDK4/6 inhibitors have improved survival, treatment response and long-term outcomes continue to vary considerably among patients, even within clinically similar HR+/HER2− metastatic breast cancer populations [2,6]. Findings from real-world multicenter studies suggest that treatment outcomes with CDK4/6 inhibitor-based therapy may vary in routine clinical practice, highlighting the heterogeneity of patients treated outside clinical trials [7]. These observations underscore the need for integrated prognostic models capable of incorporating both tumor-intrinsic and host-related determinants of outcome.

Systemic inflammation contributes to tumor progression through multiple mechanisms, including enhanced proliferation, angiogenesis, metabolic reprogramming, and immune evasion within the tumor microenvironment [8]. Serum albumin is a marker of nutrition and systemic inflammation, with lower levels linked to shorter survival in metastatic breast cancer [9]. The C-reactive protein-to-albumin ratio has also been linked to survival in patients undergoing systemic treatment [10]. Similarly, elevated neutrophil-to-lymphocyte ratio (NLR) and lactate dehydrogenase (LDH) levels have been associated with worse outcomes in HR+/HER2− metastatic breast cancer patients receiving CDK4/6 inhibitors [11]. These findings highlight the potential prognostic relevance of biochemical and host-related factors, although individual biomarkers may reflect multiple biological processes.

In addition to biochemical parameters, body composition and skeletal muscle reserve have emerged as additional determinants of oncologic prognosis [12]. Sarcopenia, characterized by progressive skeletal muscle depletion, is prevalent in advanced malignancies and has been associated with reduced survival across tumor types [12]. Baseline sarcopenia has been linked to shorter PFS and OS during CDK4/6 inhibitor treatment for HR+/HER2− metastatic breast cancer [13]. Quantitative computed tomography-based assessment of muscle mass, including skeletal muscle index (SMI), allows objective and reproducible evaluation of muscle reserve [14]. Furthermore, volumetric evaluation of bilateral psoas muscle expressed as psoas volume index (PVI) has demonstrated independent prognostic value and high reproducibility in patients with solid tumors [15]. The prognostic relevance of sarcopenic obesity has also been demonstrated in other solid tumors [16].

Tumor metabolic activity represents another central hallmark of cancer aggressiveness [17]. SUVmax reflects glucose metabolic intensity and has been associated with molecular subtype and survival in untreated metastatic breast cancer [18]. Elevated serum LDH, a key enzyme in anaerobic glycolysis, reflects tumor burden and cellular turnover and has been associated with inferior outcomes in HR+/HER2− advanced breast cancer treated with palbociclib [11]. Together, SUVmax and LDH provide complementary measures of tumor metabolic aggressiveness.

Biochemical parameters, skeletal muscle reserve parameters, and metabolic tumor indices have each demonstrated prognostic relevance in metastatic breast cancer [9,13,18]. However, each provides information about only part of the patient’s overall disease and physiological status. For example, metabolic markers do not directly account for skeletal muscle quantity, while muscle-based measures do not capture tumor metabolic activity or biochemical changes. This raises the question of whether combining these complementary parameters could provide additional prognostic information beyond individual markers. To address this gap, we aimed to develop the Metabolic–Muscle Reserve Index (MMRI), integrating SUVmax, LDH, albumin, and PVI, and assessed its relationship with progression-free survival (PFS) and overall survival (OS) in HR+/HER2− metastatic breast cancer patients receiving CDK4/6 inhibitors.

## 2. Materials and Methods

### 2.1. Study Design and Outcomes

Patients receiving CDK4/6 inhibitor therapy for HR+/HER2− metastatic breast cancer between 2020 and 2025 at Manisa Celal Bayar University Faculty of Medicine were retrospectively evaluated. The study included adults aged 18 years or older with histologically confirmed HR+/HER2− metastatic breast cancer. Those with another active malignancy or inadequate follow-up for survival analysis were excluded. Eligibility was evaluated for all patients treated consecutively during the study period, with enrollment limited to those who fulfilled the study criteria. The study was approved by the Health Sciences Ethics Committee of Manisa Celal Bayar University Faculty of Medicine (No. 20.478.486/3634; 10 December 2025) and conducted in accordance with the Declaration of Helsinki. Informed consent was waived due to the retrospective design.

Clinical, pathological, laboratory, imaging, treatment, and survival data were retrospectively retrieved from institutional medical records. Baseline variables included age, sex, body mass index (BMI), Eastern Cooperative Oncology Group (ECOG) performance status, histological subtype, metastatic presentation and sites, prior systemic therapies, treatment line, serum albumin, LDH, C-reactive protein (CRP), and CA15-3 levels. Baseline laboratory measurements were obtained within two weeks before CDK4/6 inhibitor initiation.

PFS was calculated from the first dose of a CDK4/6 inhibitor to progression or death. Disease status was reviewed by imaging approximately every 12 weeks according to RECIST 1.1. Stopping treatment was not considered progression, and patients without an event were followed until their last evaluable assessment. Overall survival was defined as the time from initiation of CDK4/6 inhibitor therapy to death from any cause. Patients who were alive at the last follow-up were censored at the date of their last clinical assessment. The data cutoff date was November 2025.

### 2.2. Image Acquisition and PET/CT Analysis

Baseline PET/CT images acquired within 15 days before CDK4/6 inhibitor initiation were retrospectively assessed. All patients fasted for at least 4–6 h prior to imaging, and blood glucose levels were confirmed to be <200 mg/dL. Each patient received an intravenous injection of 370–555 MBq (10–15 mCi) of 18F-FDG. Following a resting period of approximately 60 min in a quiet room, imaging was acquired using a General Electric Discovery IQ 3-Ring hybrid PET/CT scanner (GE Healthcare, Milwaukee, WI, USA).

A low-dose CT scan (16-slice; 120 kVp; 90 mA) was initially acquired for attenuation correction and anatomical localization. Subsequently, multibed PET acquisitions were performed in the supine position covering from the mid-thigh to the skull base (approximately 8–10 bed positions, 2 min per bed position). PET images were reconstructed using the Q.Clear penalized-likelihood reconstruction algorithm (GE Healthcare) with a beta-value of 500, a matrix size of 192 × 192, a 70 cm field of view, and a slice thickness of 3.26 mm (voxel size approx. 11.88 mm^3^). Transaxial, coronal, and sagittal images were evaluated on an Advantage Workstation (AW, GE Healthcare). For each patient, maximum standardized uptake value (SUVmax) was calculated using a three-dimensional volume of interest (VOI) placed over the lesion demonstrating the highest 18F-FDG uptake, representing the baseline peak metabolic tumor activity.

### 2.3. Assessment of Skeletal Muscle Reserve

Skeletal muscle reserve was volumetrically evaluated on the non-contrast, low-dose CT component (16-slice; 120 kVp, 90 mA) of baseline PET/CT scans using a dedicated Advantage Workstation (AW, GE Healthcare). Bilateral psoas major muscles were delineated along their craniocaudal extent, from their proximal origin at the T12-L1 level to the level of convergence with the iliacus muscle. Manual slice-by-slice freehand segmentation was performed on contiguous axial CT images by an experienced nuclear medicine specialist (with 15 years of experience in cross-sectional PET/CT imaging) who was strictly blinded to all clinical outcomes, progression status, and survival records. Skeletal muscle attenuation thresholds (−29 to +150 Hounsfield Units) were applied as a visual reference; macroscopic intramuscular adipose tissue, surrounding retroperitoneal fat, adjacent intestinal loops, and bones were meticulously excluded from muscle volume definitions. Areas with local artifacts were manually corrected. Intra-observer reproducibility was confirmed in a randomly selected representative subset of patients re-evaluated at a two-week interval, demonstrating excellent agreement (intraclass correlation coefficient > 0.90).

Bilateral psoas muscle volume was selected as a CT-derived measure of muscle quantity because three-dimensional segmentation incorporates the anatomical extent of both muscles across consecutive CT slices, rather than relying on a single axial cross-sectional measurement. Previous oncological studies have reported the feasibility and clinical relevance of volumetric psoas assessment [15,19,20]. Although the L3 skeletal muscle index (SMI) is a more established measure of skeletal muscle quantity, volumetric PVI was selected to explore its potential prognostic contribution within the MMRI. PVI was regarded as an imaging-based indicator of muscle quantity rather than a diagnostic measure of sarcopenia, because muscle strength and physical performance were not assessed.

The total volume of both psoas muscles was summed and expressed in cubic centimeters (cm^3^). Unlike the conventional L3 skeletal muscle index, which normalizes two-dimensional muscle area (cm^2^) to height squared (m^2^), PVI is derived from three-dimensional muscle volume (cm^3^). Accordingly, total bilateral psoas volume was normalized to height cubed (m^3^) to maintain dimensional consistency with the three-dimensional measurement, consistent with previously described approaches [15,20]. The resulting PVI was expressed in cm^3^/m^3^ and calculated as follows:

PVI (cm^3^/m^3^) = Total Bilateral Psoas Muscle Volume (cm^3^)/[Height (m)]^3^

The Metabolic–Muscle Reserve Index (MMRI) was calculated by combining tumor metabolic activity, circulating biochemical parameters, and CT-derived skeletal muscle quantity according to the following formula:

MMRI = [SUVmax × LDH (U/L)]/[PVI (cm^3^/m^3^) × Albumin (g/dL)]

where SUVmax represents the highest metabolic uptake of the metastatic lesion, LDH is serum lactate dehydrogenase (expressed in U/L), PVI is the psoas volume index (expressed in cm^3^/m^3^), and Albumin is serum albumin (expressed in g/dL).

The MMRI formula was developed a priori based on biological reasoning, before examining its associations with progression-free survival and overall survival. SUVmax was included as a measure of tumor metabolic activity, whereas LDH and albumin were incorporated as biochemical parameters reflecting multiple physiological and pathological processes. PVI was included as a quantitative imaging-based surrogate marker of skeletal muscle reserve. The multiplicative structure was selected to integrate metabolic activity, biochemical status, and skeletal muscle quantity into a single composite index. The formula was not adapted from an existing prognostic score or selected based on observed survival outcomes.

### 2.4. Statistical Analysis

MMRI groups were compared using Pearson’s chi-square test or Fisher’s exact test when expected counts were below five. Categorical variables were expressed as counts and percentages. Chi-square results included the test statistic, degrees of freedom, and *p*-value, whereas only *p*-values were reported for Fisher’s exact test. Survival was assessed using Kaplan–Meier curves and the log-rank test. Univariable Cox proportional hazards regression analyses were performed to evaluate potential prognostic factors. Covariates for multivariable Cox regression models were selected based on their clinical relevance, associations observed in univariable analyses, and the number of available outcome events, with the aim of limiting model complexity and reducing the risk of overfitting. Results were expressed as hazard ratios (HRs) with 95% confidence intervals (CIs). The distribution of MMRI was assessed using descriptive statistics and skewness. The relationship between continuous MMRI and survival outcomes was explored using natural cubic splines with three degrees of freedom. Given the markedly right-skewed distribution of MMRI, a natural logarithmic transformation was evaluated. Model fit was compared using the Akaike information criterion (AIC). Natural log-transformed MMRI was used in both univariable and multivariable Cox regression analyses because it provided better model fit than untransformed MMRI.

Natural cubic spline analyses and Schoenfeld residual-based tests for the proportional hazards assumption were performed using R software (version 4.5.3; R Foundation for Statistical Computing, Vienna, Austria). Missing data were handled using a complete-case approach without imputation. Patients with missing baseline variables required for MMRI calculation were excluded from MMRI-based analyses. For multivariable Cox regression analyses, only patients with complete data for all covariates included in the respective models were analyzed.

To compare the prognostic discrimination of MMRI with that of its individual components (SUVmax, LDH, albumin, and PVI), Harrell’s concordance indices (C-indices) and corresponding standard errors were calculated in a common complete-case cohort of 163 patients. The incremental prognostic value of MMRI beyond established clinical factors was assessed by comparing nested Cox proportional hazards models with and without log-transformed MMRI using likelihood-ratio tests and Harrell’s C-index. Internal validation was performed using 1000 bootstrap resamples to estimate optimism-corrected C-indices for the clinical models with and without MMRI. These additional prognostic analyses were performed using R statistical software.

An exploratory MMRI threshold was determined using Youden’s index from a ROC analysis based on mortality status at the last follow-up. Discrimination was assessed by the area under the curve (AUC). To examine the ability of MMRI to predict overall survival at 12, 24, and 36 months, time-dependent ROC analyses were conducted in R, applying inverse probability of censoring weighting to account for censored data. The remaining analyses were carried out in IBM SPSS Statistics for Windows, version 27.0 (IBM Corp., Armonk, NY, USA). Statistical significance was defined as a two-sided *p*-value < 0.05.

## 3. Results

### 3.1. Patient Characteristics

**Figure 1:**
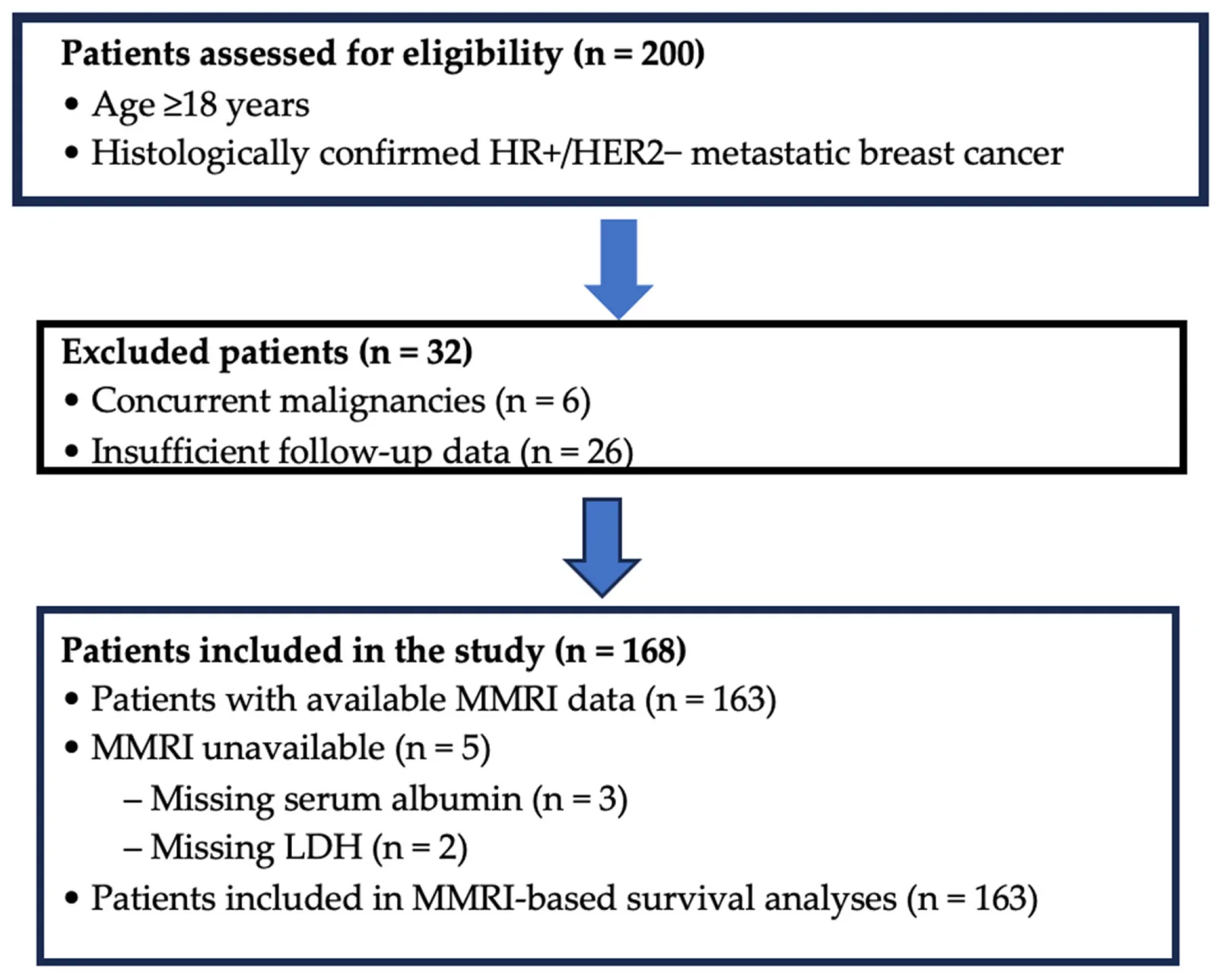
Flowchart of patient inclusion and study cohort formation.

We reviewed the records of 200 individuals who received CDK4/6 inhibitors for HR+/HER2− metastatic breast cancer. Among them, 32 were excluded because of another malignancy (n = 6) or inadequate follow-up for survival analysis (n = 26), leaving 168 patients in the study. MMRI was unavailable for five patients (3.0%) due to missing baseline albumin (n = 3) or LDH (n = 2) values. Therefore, MMRI-based survival analyses included 163 patients (97.0%). Figure 1 illustrates the patient selection process.

The median age was 55 years (26–81), and 97.6% of the cohort were female. ECOG performance status was 0–1 in 88.7% of patients. Invasive ductal carcinoma was the predominant histological subtype (90.5%). At diagnosis, 41.1% of patients had de novo metastatic disease, while 58.9% developed metastases after an earlier breast cancer diagnosis. Bone was the most frequently affected site (78.6%), followed by lymph nodes (59.5%) and visceral organs (58.3%). Lung and liver involvement occurred in 39.3% and 26.8% of patients, respectively, while brain metastases were less frequent (5.4%).

Most patients (78.0%) received CDK4/6 inhibitors as their first-line systemic therapy. Ribociclib and palbociclib were administered in 48.8% and 51.2% of cases, respectively. Concomitant endocrine therapy consisted of letrozole in 61.3% and fulvestrant in 38.7%. Prior endocrine therapy before CDK4/6 initiation had been administered in 22.0% of patients, and 31.5% had received prior chemotherapy. Dose reduction of the CDK4/6 inhibitor occurred in 35.7% of cases. Grade 1–2 and grade 3–4 adverse events were reported in 48.2% and 42.3% of patients, respectively. Subsequent systemic therapy following CDK4/6 inhibitor discontinuation was administered in 47.7% of patients.

The median SUVmax was 11.20 (1.40–34.90). The median total psoas volume was 244.50 (102.70–490.60), and the median psoas volume index was 60.56 (26.04–105.96). Receiver operating characteristic curve analysis for mortality established 10.68 as the optimal MMRI threshold with an AUC of 0.642 (95% CI: 0.563–0.716), indicating modest discriminative performance. At this cut-off, sensitivity and specificity were 61.97% and 60.87%, respectively. Using this cutoff, patients were categorized into MMRI ≤ 10.68 and MMRI > 10.68 groups for exploratory survival analyses. Between-group comparisons revealed significant differences in metastatic presentation pattern (*p* = 0.024), bone involvement (*p* = 0.003), lymph node metastasis (*p* = 0.002), prior chemotherapy exposure before CDK4/6 inhibitor initiation (*p* = 0.043), and mortality (*p* = 0.004), as presented in Table 1.

### 3.2. MMRI Distribution and Model Assessment

MMRI exhibited a markedly right-skewed distribution (skewness = 10.24), which was substantially reduced after natural logarithmic transformation (skewness = 0.38). In multivariable Cox regression models, natural log-transformed MMRI [ln(MMRI)] yielded lower Akaike information criterion (AIC) values than untransformed MMRI for both PFS (667.83 vs. 674.00) and OS (604.65 vs. 610.06). Accordingly, ln(MMRI) was used as a continuous predictor in subsequent univariable and multivariable Cox regression analyses.

### 3.3. Univariate and Multivariate Analyses of Progression-Free Survival and Overall Survival

Among the 163 patients included in the MMRI-based survival analyses, the median follow-up duration was 39.6 months (95% CI, 34.7–43.9). During follow-up, 101 PFS events (62.0%) and 71 deaths (43.6%) were recorded.

In univariate analysis, liver (HR, 1.989; 95% CI, 1.327–2.981; *p* = 0.001), lymph node (HR, 1.877; 95% CI, 1.247–2.824; *p* = 0.003), and brain metastases (HR, 3.258; 95% CI, 1.620–6.551; *p* = 0.001) were associated with shorter PFS. High proliferative activity (Ki-67 > 50%; HR, 2.040; 95% CI, 1.127–3.695; *p* = 0.019), prior endocrine therapy before CDK4/6 inhibitor initiation (HR, 1.811; 95% CI, 1.195–2.746; *p* = 0.005), prior chemotherapy exposure (HR, 1.600; 95% CI, 1.081–2.367; *p* = 0.019), and increasing CDK4/6 treatment line (HR, 1.195; 95% CI, 1.040–1.373; *p* = 0.011) also emerged as significant predictors of shorter PFS. Among laboratory variables, elevated LDH (HR, 1.001; 95% CI, 1.001–1.002; *p* < 0.001) and increasing MMRI values (HR, 1.988; 95% CI, 1.504–2.629; *p* < 0.001) were associated with poorer PFS. Patients with an MMRI > 10.68 had a nearly twofold higher risk of progression (HR, 1.941; 95% CI, 1.301–2.895; *p* = 0.001).

In multivariate analysis, liver metastasis (HR, 1.746; 95% CI, 1.099–2.774; *p* = 0.018), lymph node metastasis (HR, 1.683; 95% CI, 1.005–2.819; *p* = 0.047), brain metastasis (HR, 2.854; 95% CI, 1.205–6.758; *p* = 0.017), and MMRI (HR, 1.857; 95% CI, 1.329–2.596; *p* = 0.0003) remained significantly associated with shorter PFS (Table 2). Prior endocrine therapy and CDK4/6 treatment line were included in the adjusted model but were not independently associated with PFS.

In univariate Cox regression analysis, liver (HR, 1.838; 95% CI, 1.148–2.943; *p* = 0.011), bone (HR, 2.245; 95% CI, 1.179–4.274; *p* = 0.014), lymph node (HR, 1.806; 95% CI, 1.107–2.947; *p* = 0.018), and brain metastases (HR, 3.743; 95% CI, 1.782–7.862; *p* < 0.001) were associated with shorter OS. Patients who had received prior endocrine therapy also had shorter OS (HR, 2.007; 95% CI, 1.253–3.215; *p* = 0.004), as did those receiving CDK4/6 inhibitors in later treatment lines (HR, 1.254; 95% CI, 1.070–1.470; *p* = 0.005).

Among laboratory parameters, higher LDH (HR, 1.002; 95% CI, 1.001–1.002; *p* < 0.001) and CA 15-3 levels (HR, 1.001; 95% CI, 1.000–1.001; *p* = 0.001), as well as lower albumin levels (HR, 0.410; 95% CI, 0.230–0.730; *p* = 0.002), were associated with shorter OS. Higher MMRI was also associated with shorter OS (HR, 2.296; 95% CI, 1.634–3.226; *p* < 0.001). Patients with MMRI > 10.68 had more than twice the risk of death compared with those with MMRI ≤ 10.68 (HR, 2.205; 95% CI, 1.363–3.566; *p* = 0.001).

In multivariable analysis, liver metastasis (HR, 1.652; 95% CI, 1.009–2.705; *p* = 0.046), brain metastasis (HR, 3.111; 95% CI, 1.410–6.864; *p* = 0.004), and MMRI (HR, 1.974; 95% CI, 1.368–2.846; *p* = 0.0003) remained independently associated with shorter OS (Table 3). Prior endocrine therapy and CDK4/6 treatment line were not statistically significant in the adjusted OS model.

Natural cubic spline analyses of log-transformed MMRI did not demonstrate a significant improvement in model fit compared with the corresponding linear models for either PFS (χ^2^ = 0.645, df = 2, *p* = 0.724) or OS (χ^2^ = 0.832, df = 2, *p* = 0.660). The proportional hazards assumption was assessed using Schoenfeld residuals. No statistically significant violations were detected for any individual covariate or in the global tests for either the multivariable PFS model (χ^2^ = 7.672, df = 8, *p* = 0.470) or OS model (χ^2^ = 6.601, df = 7, *p* = 0.472) (Supplementary Table S1).

**Table 2:** Univariate and Multivariate Cox Regression Analyses for Progression-Free Survival.

	Univariate			Multivariate		
	HR	95% CI	*p*	HR	95% CI	*p*
Age	0.991	0.976–1.006	0.223			
Sex	2.261	0.827–6.178	0.112			
Comorbidity	0.849	0.579–1.246	0.404			
BMI	0.982	0.947–1.017	0.308			
ECOG			0.529			
1 vs. 0	1.065	0.711–1.596	0.758			
2 vs. 0	0.704	0.347–1.426	0.330			
Histology (Ref: invasive ductal) invasive lobular			0.999			
	1.126	0.547–2.319	0.747			
Metastatic pattern (Ref: de novo metastatic)	1.355	0.909–2.020	0.136			
Adjuvant chemotherapy	1.269	0.865–1.863	0.223			
Adjuvant hormonotherapy	0.498	0.197–1.258	0.140			
Ki-67			0.062			
20–50% vs. 1–20% >50% vs. 1–20%	1.240	0.767–2.003	0.381	1.204	0.715–2.026	0.485
	2.040	1.127–3.695	0.019	1.247	0.672–2.313	0.483
Liver metastasis	1.989	1.327–2.981	0.001	1.746	1.099–2.774	0.018
Bone metastasis	1.497	0.925–2.421	0.100			
Lung metastasis	1.232	0.835–1.816	0.293			
Lymph node metastasis	1.877	1.247–2.824	0.003	1.683	1.005–2.819	0.047
Brain metastasis	3.258	1.620–6.551	0.001	2.854	1.205–6.758	0.017
Visceral metastasis	2.044	1.357–3.080	0.001			
SUVmax	1.056	1.025–1.088	<0.001			
Total psoas volume	0.999	0.996–1.002	0.626			
Left psoas volume	0.997	0.991–1.004	0.422			
Right psoas volume	0.999	0.994–1.005	0.850			
Menopausal status			0.277			
Postmenopausal vs. premenopausal	1.046	0.669–1.635	0.845			
Prior endocrine therapy before CDK4/6	1.811	1.195–2.746	0.005	1.536	0.743–3.177	0.247
Prior chemotherapy before CDK4/6	1.600	1.081–2.367	0.019			
CDK4/6 treatment line	1.195	1.040–1.373	0.011	1.118	0.867–1.442	0.391
Number of prior chemotherapy lines	0.986	0.702–1.384	0.934			
Type of CDK4/6 inhibitor (Ref: ribociclib)	1.277	0.866–1.883	0.216			
Concomitant endocrine therapy (Ref: letrozole)	1.022	0.689–1.517	0.914			
PVI	0.997	0.984–1.011	0.672			
CRP	1.019	0.940–1.105	0.642			
LDH	1.001	1.001–1.002	<0.001			
Albumin	0.745	0.448–1.241	0.259			
CA 15-3	1.001	1.000–1.001	0.050			
MMRI *	1.988	1.504–2.629	<0.001	1.857	1.329–2.596	0.0003

* MMRI was natural log-transformed before inclusion in the Cox regression model. The hazard ratio for MMRI (HR, 1.857) indicates that a one-unit increase in ln(MMRI) was associated with an 85.7% higher hazard of progression. Abbreviations: HR, hazard ratio; CI, confidence interval; BMI, body mass index; ECOG, Eastern Cooperative Oncology Group; SUVmax, maximum standardized uptake value; CDK4/6, cyclin-dependent kinase 4/6; MMRI, Metabolic–Muscle Reserve Index.

**Table 3:** Univariate and Multivariate Cox Regression Analyses for Overall Survival.

	Univariate			Multivariate		
	HR	95% CI	*p*	HR	95% CI	*p*
Age	1.001	0.983–1.020	0.902			
Sex	2.432	0.886–6.678	0.085			
Comorbidity	0.869	0.550–1.373	0.548			
BMI	0.988	0.947–1.031	0.577			
ECOG			0.604			
1 vs. 0 2 vs. 0	1.217	0.747–1.983	0.430			
	1.365	0.656–2.843	0.405			
Histology (Ref: invasive ductal) Invasive lobular			0.850			
	1.595	0.728–3.491	0.243			
Metastatic pattern (Ref: de novo metastatic)	1.338	0.822–2.177	0.242			
Adjuvant chemotherapy	0.980	0.621–1.547	0.931			
Adjuvant hormonotherapy	1.705	0.412–7.061	0.461			
Ki-67 20–50% vs. 1–20% >50% vs. 1–20%			0.549			
	0.758	0.422–1.361	0.353			
	1.081	0.519–2.252	0.834			
Liver metastasis	1.838	1.148–2.943	0.011	1.652	1.009–2.705	0.046
Bone metastasis	2.245	1.179–4.274	0.014	2.047	0.995–4.212	0.051
Lung metastasis	1.236	0.778–1.965	0.369			
Lymph node metastasis	1.806	1.107–2.947	0.018	1.554	0.893–2.705	0.118
Brain metastasis	3.743	1.782–7.862	<0.001	3.111	1.410–6.864	0.004
Visceral metastasis	1.770	1.081–2.897	0.023			
SUVmax	1.049	1.011–1.088	0.012			
Menopausal status postmenopausal vs. premenopausal			0.155			
	1.289	0.737–2.253	0.373			
Prior endocrine therapy before CDK4/6	2.007	1.253–3.215	0.004	1.637	0.833–3.215	0.153
Prior chemotherapy before CDK4/6	1.569	0.989–2.487	0.056			
CDK4/6 treatment line	1.254	1.070–1.470	0.005	1.205	0.943–1.541	0.135
Number of prior chemotherapy lines	1.195	0.848–1.685	0.309			
Type of CDK4/6 inhibitor (Ref: ribociclib)	1.199	0.751–1.916	0.447			
Concomitant endocrine therapy (Ref: letrozole)	1.062	0.666–1.694	0.801			
Total psoas volume	0.999	0.995–1.003	0.588			
Left psoas volume	0.996	0.989–1.004	0.347			
Right psoas volume	0.999	0.993–1.006	0.871			
Psoas volume index	0.995	0.978–1.011	0.525			
Psoas volume index > 49.375	1.157	0.574–2.331	0.684			
CRP	1.070	0.981–1.167	0.126			
LDH	1.002	1.001–1.002	<0.001			
Albumin	0.410	0.230–0.730	0.002			
CA 15-3	1.001	1.000–1.001	0.001			
MMRI *	2.296	1.634–3.226	<0.001	1.974	1.368–2.846	0.0003

* MMRI was natural log-transformed before inclusion in the Cox regression model. The hazard ratio for MMRI (HR, 1.974) indicates that a one-unit increase in ln(MMRI) was associated with a 97.4% higher hazard of death. HR, hazard ratio; CI, confidence interval; BMI, body mass index; ECOG, Eastern Cooperative Oncology Group; SUVmax, maximum standardized uptake value; CDK4/6, cyclin-dependent kinase 4/6; MMRI, Metabolic–Muscle Reserve Index.

### 3.4. Survival Outcomes

**Figure 2:**
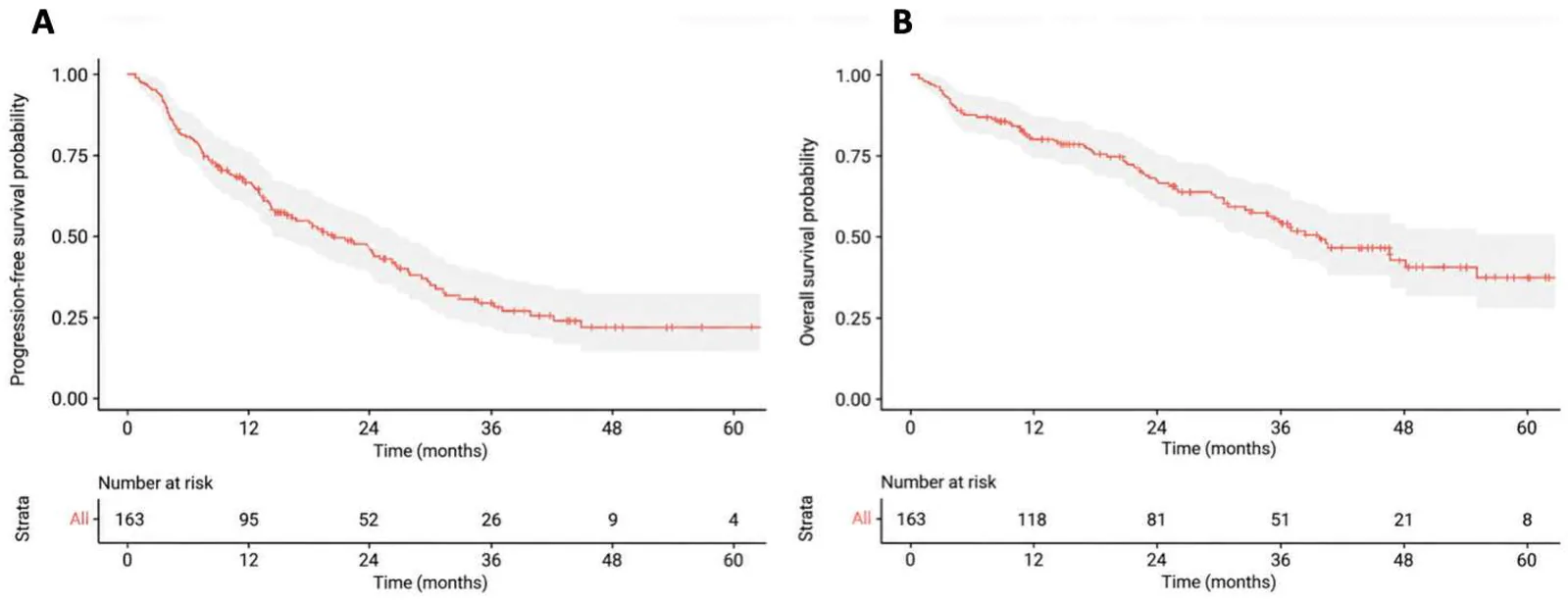
Kaplan–Meier survival estimates for the overall study population. (**A**) Progression-free survival (PFS). (**B**) Overall survival (OS).

**Figure 3:**
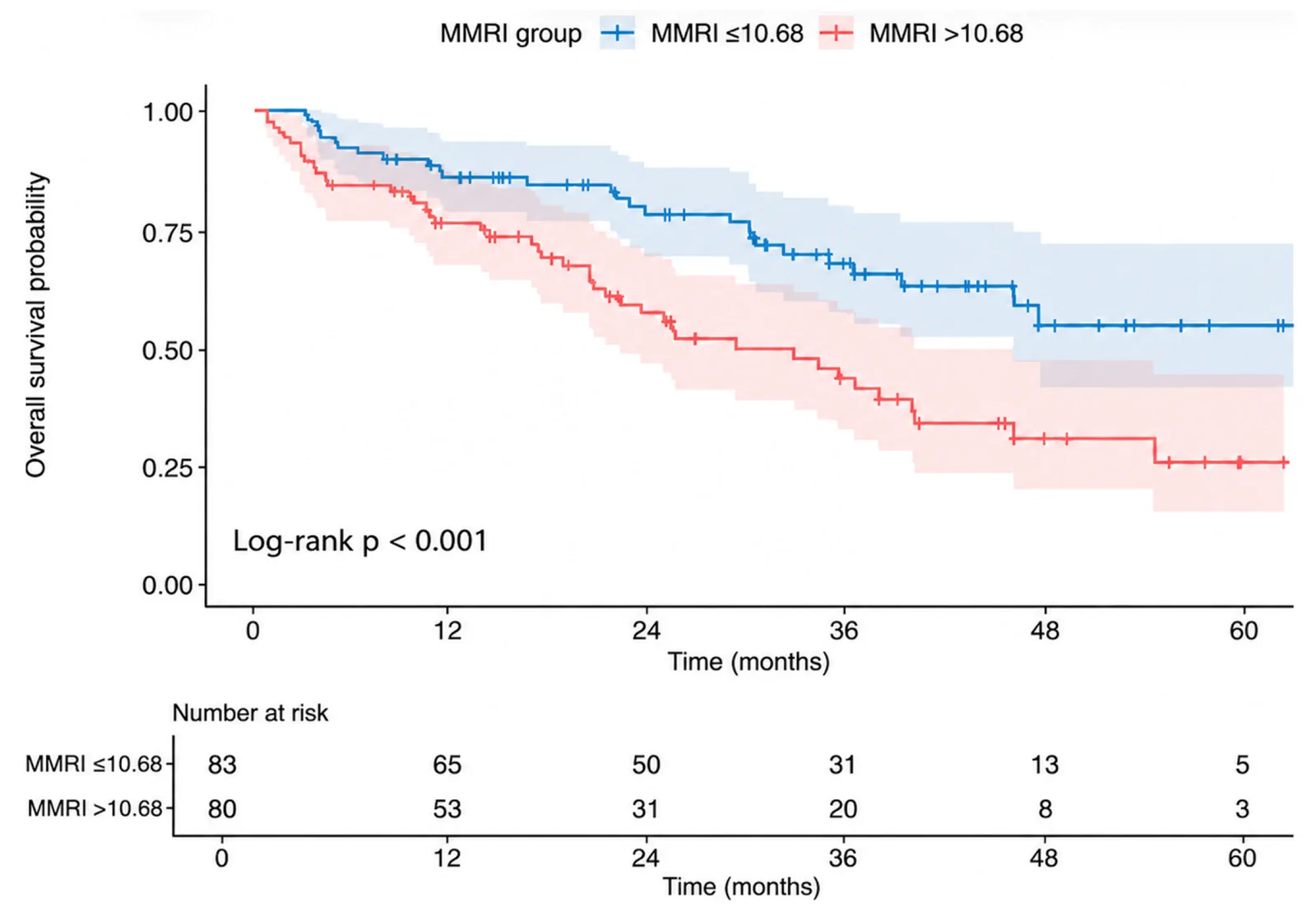
Kaplan–Meier analysis of overall survival stratified by the Metabolic–Muscle Reserve Index (MMRI). Shaded areas represent 95% confidence intervals, and numbers at risk are displayed below the survival curves. Median OS and its 95% CI were not reached in the MMRI ≤ 10.68 group.

In the overall patient population (n = 168) the median PFS was 20.4 months, with 1-, 3-, and 5-year PFS rates of 64.5%, 28.1%, and 22.3%, respectively (Figure 2A). Median OS was 39.8 months (SE, 5.0; 95% CI, 30.1–49.5), with corresponding survival rates of 80.5%, 53.5%, and 37.3% (Figure 2B). MMRI-stratified Kaplan–Meier survival analyses were performed in the 163 patients with available data. When patients were grouped according to the exploratory, data-derived MMRI cut-off of 10.68, statistically significant differences in survival outcomes were observed. Patients with MMRI > 10.68 had shorter OS compared with those with MMRI ≤ 10.68 (median OS: 29.7 months vs. not reached; log-rank *p* < 0.001). The 1-, 3-, and 5-year OS rates were 75.7%, 41.9%, and 24.4% in the MMRI > 10.68 group and 85.3%, 66.7%, and 53.3% in the MMRI ≤ 10.68 group, respectively (Figure 3).

**Figure 4:**
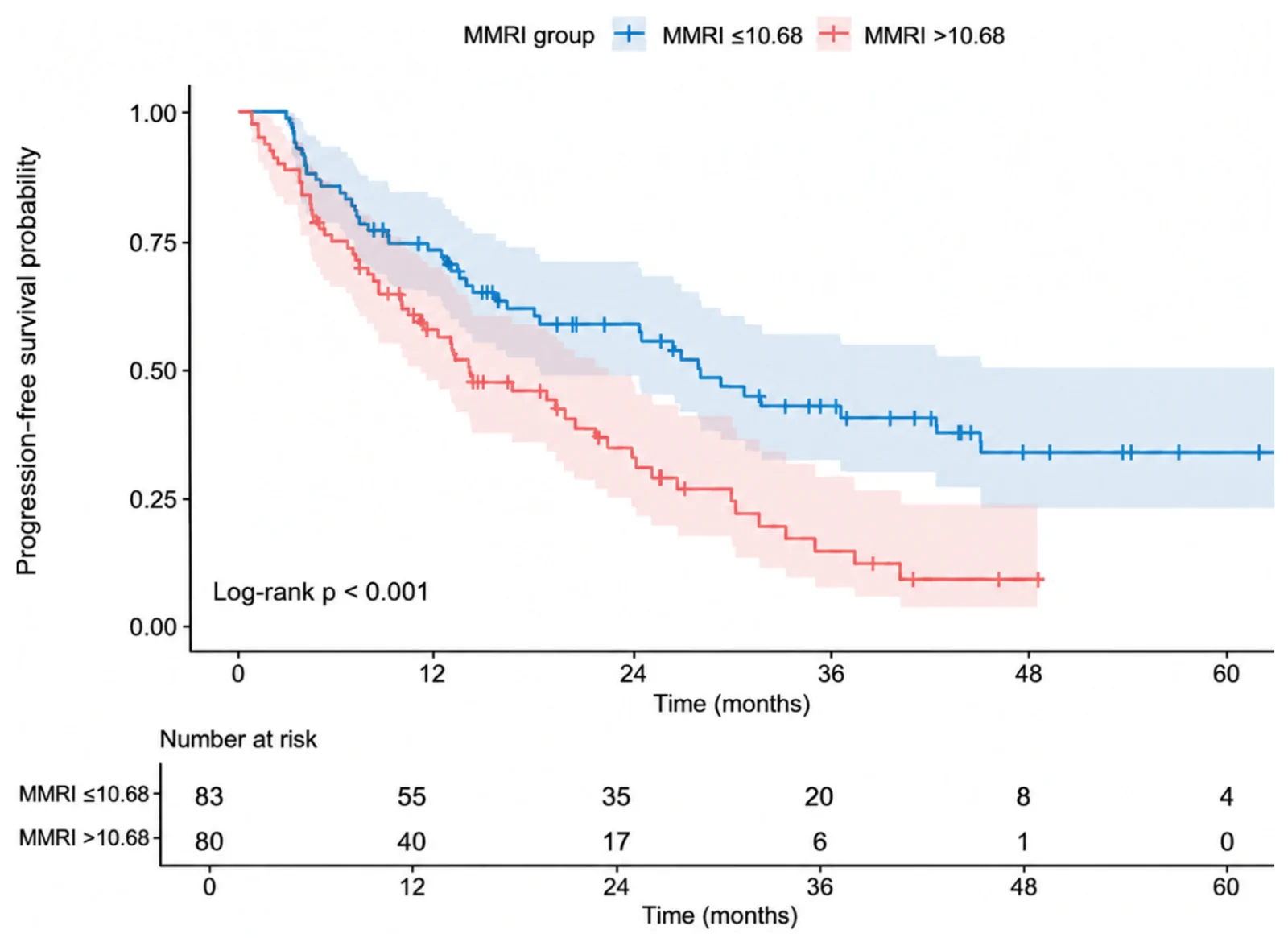
Kaplan–Meier analysis of progression-free survival stratified by the Metabolic–Muscle Reserve Index (MMRI). Shaded areas represent 95% confidence intervals, and numbers at risk are displayed below the survival curves.

Progression-free survival also differed between MMRI groups. Median PFS was 14.1 months in patients with MMRI > 10.68 and 28.1 months in those with MMRI ≤ 10.68 (log-rank *p* < 0.001). The 1- and 3-year PFS rates were 57.1% and 14.9% in the MMRI > 10.68 group and 71.9% and 40.8% in the MMRI ≤ 10.68 group, respectively (Figure 4). The 5-year PFS rate was 34.1% in the MMRI ≤ 10.68 group, whereas the corresponding estimate was not available in the MMRI > 10.68 group, with no patients remaining at risk at five years.

Additionally, time-dependent ROC analysis demonstrated modest discriminative performance of MMRI for overall survival, with AUCs of 0.672 (95% CI: 0.563–0.781), 0.681 (95% CI: 0.583–0.780), and 0.663 (95% CI: 0.562–0.764) at 12, 24, and 36 months, respectively.

### 3.5. Additional Prognostic Contribution of MMRI Beyond Clinical Factors

To compare the prognostic discrimination of MMRI with that of its individual components, Harrell’s concordance indices were calculated in a common complete-case cohort of 163 patients. MMRI yielded the highest numerical C-index for both OS (0.652; SE, 0.038) and PFS (0.625; SE, 0.033). For OS, the corresponding C-indices were 0.565 for SUVmax, 0.602 for LDH, 0.617 for albumin, and 0.535 for PVI. For PFS, the respective values were 0.591, 0.565, 0.548, and 0.536 (Supplementary Table S2). Adding MMRI to the clinical models significantly improved model fit for both PFS (likelihood-ratio χ^2^ = 12.254, *p* < 0.001) and OS (χ^2^ = 13.115, *p* < 0.001). Harrell’s C-index increased modestly from 0.678 to 0.700 for PFS and from 0.698 to 0.721 for OS. Following bootstrap internal validation, the optimism-corrected C-indices of the MMRI-extended models were 0.671 for PFS and 0.704 for OS (Supplementary Table S3).

## 4. Discussion

The present study demonstrates that the Metabolic–Muscle Reserve Index, a composite parameter integrating tumor metabolic activity, biochemical status, and CT-derived skeletal muscle quantity, is independently associated with both PFS and OS among patients receiving CDK4/6 inhibitor therapy for HR+/HER2− metastatic breast cancer. These findings support the concept that both tumor-related and host-related factors contribute to outcome variability in this setting and highlight the potential value of multidimensional prognostic approaches.

Biochemical and nutritional parameters have been increasingly investigated as practical, baseline predictors of benefit from CDK4/6 inhibitor-based therapy in metastatic breast cancer [21].

In a single-center cohort of patients receiving palbociclib combined with endocrine therapy, commonly available blood markers, including LDH and albumin, were examined in relation to progression outcomes [21]. Integrated prognostic models that combine inflammatory, nutritional, and sarcopenia-related domains have further emphasized the prognostic relevance of host status in metastatic breast cancer [22]. Albumin-derived ratios may further refine risk estimation, as the albumin-to-globulin ratio has also been investigated as a prognostic marker in breast cancer [23]. A recent study linked higher LDH-to-albumin ratios to shorter PFS and OS in patients receiving CDK4/6 inhibitors. This supports the prognostic role of biochemical markers included in MMRI [24].

Tumor metabolic activity has also been associated with prognosis in metastatic breast cancer, and FDG PET/CT provides a noninvasive method for assessing this biological characteristic [18]. In previously untreated metastatic breast cancer, baseline SUVmax was associated with survival and correlated with disease characteristics, supporting its prognostic value [18]. More recently, pretreatment FDG PET/CT has also been investigated as a prognostic imaging biomarker in patients receiving CDK4/6 inhibitors [25].

Body composition has attracted increasing attention as a factor that may influence outcomes during targeted therapy. In patients with HR+/HER2− metastatic breast cancer receiving CDK4/6 inhibitors, baseline body composition parameters, including skeletal muscle measurements, have been evaluated in relation to treatment response and survival, suggesting that sarcopenia and obesity provide clinically relevant prognostic information [14]. This observation is supported by a recent systematic review and meta-analysis showing that sarcopenia is consistently associated with poorer survival in metastatic breast cancer [26]. Other CT-derived muscle measurements have also demonstrated prognostic value. In patients with breast cancer and bone metastases, lower psoas muscle density was associated with shorter overall survival, further supporting the clinical relevance of imaging-based muscle assessment [27].

The method used to assess skeletal muscle quantity may influence its prognostic relevance. Previous studies have investigated three-dimensional psoas muscle volume in patients with solid tumors, demonstrating the feasibility and potential clinical relevance of volumetric assessment [15,19,20]. In contrast to the conventional L3 skeletal muscle index (SMI), which evaluates the cross-sectional area of multiple skeletal muscles at a single anatomical level, volumetric psoas assessment captures muscle quantity across the segmented region. However, these approaches are not interchangeable, as psoas volume reflects a specific muscle group rather than overall skeletal muscle quantity. Accordingly, the use of PVI in the present study should be interpreted as an exploratory approach to incorporating imaging-derived muscle quantity into MMRI rather than evidence of superiority over L3 SMI. Single-slice L3-SMI measurements may be influenced by anatomical variability, slice selection, and patient positioning. In contrast, three-dimensional bilateral psoas segmentation incorporates the anatomical extent of the muscles across consecutive CT slices, potentially reducing dependence on a single anatomical level. However, whether this approach provides superior reproducibility or prognostic performance compared with conventional L3-SMI remains uncertain and requires direct comparative validation.

These findings suggest that combining tumor metabolic activity, biochemical parameters, and skeletal muscle quantity may provide a broader assessment of prognosis in metastatic breast cancer. In our study, MMRI brings together SUVmax, LDH, albumin, and PVI to reflect both tumor-related and patient-related factors.

Our study has several strengths. First, MMRI is based on routinely available imaging and laboratory parameters, which may facilitate its evaluation in future clinical studies. Notably, bilateral psoas volume was assessed using the CT component of existing baseline PET/CT examinations, without requiring additional imaging or radiation exposure beyond the original examination. Second, it combines tumor metabolic activity, biochemical status, and CT-derived skeletal muscle quantity, allowing different aspects of tumor and patient characteristics to be considered together. MMRI remained significantly associated with both PFS and OS after adjustment for relevant clinicopathologic factors. Survival differences were also observed using the exploratory, data-derived cut-off of 10.68. These findings support further investigation of MMRI as a potential prognostic marker in patients receiving CDK4/6 inhibitor-based therapy.

Our study has several limitations. Its retrospective nature may have led to selection bias and prevents causal conclusions. Endocrine resistance could not be reliably assessed retrospectively, and residual confounding cannot be excluded despite adjustment for prior endocrine therapy and treatment line. Because all patients received CDK4/6 inhibitors, we could not determine whether MMRI predicts benefit specifically from these agents. The observed associations should therefore be interpreted as prognostic rather than treatment-specific predictive findings.

The MMRI analysis cohort included 101 PFS events and 71 deaths. The multivariable Cox models estimated eight parameters for PFS and seven for OS, corresponding to approximately 12.6 and 10.1 events per parameter, respectively. Although these event counts permitted multivariable analyses, the relatively limited number of events, particularly for OS, may have affected the stability and precision of some estimates. This is reflected in the wider confidence intervals observed for certain clinical variables. These results should be viewed with caution and validated in larger patient cohorts.

The MMRI cut-off was established and evaluated in the same cohort, which may have led to overfitting and an overestimation of the observed survival differences. The AUC of 0.642 indicates modest discriminative ability, with time-dependent ROC analyses showing similar results across the evaluated time points. The small number of patients remaining at risk at five years also limits the precision of long-term survival estimates, particularly in the higher MMRI group. These findings should therefore be considered exploratory, and the proposed cut-off requires validation in larger, independent cohorts before it can be used for clinical risk stratification.

Albumin and LDH may be affected by inflammatory processes but are not specific markers of systemic inflammation. MMRI should therefore be viewed as a composite measure of tumor metabolic activity, biochemical status, and skeletal muscle quantity rather than a direct indicator of systemic inflammation. Changes in these parameters during treatment were not assessed, and whether serial MMRI measurements provide additional prognostic information remains unknown.

The assessment of skeletal muscle quantity also has limitations. Although bilateral psoas volume has been examined in previous oncological studies, PVI cannot be considered equivalent to the more established L3 skeletal muscle index, and the two measures were not directly compared in our cohort [15,19,20]. The use of height-cubed normalization has been reported previously, but whether it provides the most appropriate adjustment for body size remains uncertain [20]. Muscle strength and physical performance were not evaluated, preventing the application of accepted diagnostic criteria for sarcopenia [28]. Accordingly, PVI reflects imaging-derived psoas muscle quantity and should not be interpreted as a measure of clinically diagnosed sarcopenia. Although intraobserver reproducibility was assessed in a subset of patients and demonstrated excellent agreement (ICC > 0.90), interobserver reproducibility was not evaluated because measurements were performed by a single trained nuclear medicine physician. Therefore, measurement variability between different observers remains uncertain, potentially limiting the generalizability of the volumetric assessment. Future studies should incorporate standardized measurement protocols and evaluate both intraobserver and interobserver reproducibility in larger cohorts.

In summary, MMRI represents a novel composite prognostic index based on tumor metabolic activity, biochemical parameters, and skeletal muscle quantity. Its association with both PFS and OS suggests that it may provide useful prognostic information in patients receiving CDK4/6 inhibitor-based therapy. However, further studies are needed to establish its clinical relevance.

## 5. Conclusions

We developed a prognostic index combining PET/CT metabolic findings, CT-based muscle measurements, and routine laboratory markers to assess outcomes during CDK4/6 inhibitor treatment for HR+/HER2− metastatic breast cancer. Higher MMRI values were associated with shorter PFS and OS. However, its modest discriminative performance, the exploratory nature of the proposed cut-off, and the retrospective single-center design warrant cautious interpretation. These findings suggest a prognostic association but do not establish that MMRI predicts benefit specifically from CDK4/6 inhibitors. Validation in larger prospective multicenter studies is required before MMRI can be considered for routine clinical use.

## Figures and Tables

**Table 1:** Demographic and Clinical Characteristics of Patients.

			MMRI			
		Total	≤10.68	>10.68	χ^2^ (df)	*p*
		n (%)	n (%)	n (%)		
Age Mean ± SD/(Min–Max)		55.28 ± 12.11 (26–81)	54.22 ± 12.23 (30–81)	56.36 ± 11.81 (26–80)		0.317
Sex						0.361
	Male	4 (2.4)	1 (1.2)	3 (3.8)		
	Female	164 (97.6)	82 (98.8)	77 (96.3)		
Comorbidity						0.705
	No	80 (47.6)	38 (45.8)	39 (48.8)		
	Yes	88 (52.4)	45 (54.2)	41 (51.2)		
ECOG					0.476 (2)	0.785
	0	84 (50.0)	43 (51.8)	38 (47.5)		
	1	65 (38.7)	32 (38.6)	32 (40.0)		
	2	19 (11.3)	8 (9.6)	10 (12.5)		
Histology						1.000
	Invasive ductal carcinoma	152 (90.5)	74 (89.2)	73 (91.3)		
	Invasive lobular carcinoma	13 (7.7)	6 (7.2)	7 (8.8)		
	Other	3 (1.8)	3 (3.6)	0 (0.0)		
Metastatic Pattern					5.119 (1)	0.024
	De novo	69 (41.1)	28 (33.7)	41 (51.2)		
	Recurrence	99 (58.9)	55 (66.3)	39 (48.8)		
Ki-67					0.332 (2)	0.847
	1–20%	77 (45.8%)	40 (48.2%)	35 (43.8%)		
	20–50%	63 (37.5%)	30 (36.1%)	31 (38.8%)		
	>%50	28 (16.7%)	13 (15.7%)	14 (17.5%)		
Adjuvant chemotherapy					3.276 (1)	0.070
	No	88 (52.4)	37 (44.6)	47 (58.8)		
	Yes	80 (47.6)	46 (55.4)	33 (41.3)		
Adjuvant hormonotherapy						0.040
	No	7 (7.1)	1 (1.8)	6 (15.4)		
	Yes	92 (92.9)	54 (98.2)	33 (84.6)		
Liver metastasis					3.030 (1)	0.082
	No	123 (73.2)	66 (79.5)	54 (67.5)		
	Yes	45 (26.8)	17 (20.5)	26 (32.5)		
Bone metastasis					8.787 (1)	0.003
	No	36 (21.4)	25 (30.1)	9 (11.3)		
	Yes	132 (78.6)	58 (69.9)	71 (88.8)		
Lung metastasis					0.982 (1)	0.339
	No	102 (60.7)	54 (65.1)	46 (57.5)		
	Yes	66 (39.3)	29 (34.9)	34 (42.5)		
Lymph node metastasis					9.905 (1)	0.002
	No	68 (40.5)	44 (53.0)	23 (28.7)		
	Yes	100 (59.5)	39 (47.0)	57 (71.3)		
Brain metastasis						0.743
	No	159 (94.6)	79 (95.2)	75 (93.8)		
	Yes	9 (5.4)	4 (4.8)	5 (6.3)		
Visceral metastasis					4.047 (1)	0.057
	No	70 (41.7)	42 (50.6)	28 (35.0)		
	Yes	98 (58.3)	41 (49.4)	52 (65.0)		
Menopausal Status					0.799 (1)	0.375
Premenopausal		41 (24.4)	22 (26.5)	16 (20.0)		
Postmenopausal		123 (73.2)	60 (72.3)	61 (76.3)		
Prior endocrine therapy before CDK4/6					0.098 (1)	0.754
	No	131 (78.0)	66 (79.5)	62 (77.5)		
	Yes	37 (22.0)	17 (20.5)	18 (22.5)		
Type of prior endocrine therapy *						1.000
	Aromatase inhibitor	16 (39.0)	8 (42.1)	7 (36.8)		
	Tamoxifen	1 (2.4)	0 (0.0)	1 (5.3)		
	Fulvestrant	24 (58.5)	11 (57.9)	11 (57.9)		
Prior chemotherapy before CDK4/6						0.043
	No	115 (68.5)	63 (75.9)	48 (60.0)		
	Yes	53 (31.5)	20 (24.1)	32 (40.0)		
Number of prior chemotherapy lines						0.860
	1	38 (71.7)	14 (70.0)	23 (71.9)		
	2	7 (13.2)	2 (10.0)	5 (15.6)		
	3	6 (11.3)	3 (15.0)	3 (9.4)		
	4	2 (3.8)	1 (5.0)	1 (3.1)		
Type of CDK4/6 inhibitor					0.162 (1)	0.688
	Ribociclib	82 (48.8)	41 (49.4)	37 (46.3)		
	Palbociclib	86 (51.2)	42 (50.6)	43 (53.8)		
Concomitant endocrine therapy					1.198 (1)	0.274
	Letrozole	103 (61.3)	47 (56.6)	52 (65.0)		
	Fulvestrant	65 (38.7)	36 (43.4)	28 (35.0)		
Grade 1–2 adverse effect					0.004 (1)	0.948
	No	87 (51.8)	44 (53.0)	42 (52.5)		
	Yes	81 (48.2)	39 (47.0)	38 (47.5)		
Grade 3–4 adverse effect					0.458 (1)	0.498
	No	97 (57.7)	50 (60.2)	44 (55.0)		
	Yes	71 (42.3)	33 (39.8)	36 (45.0)		
CDK4/6 dose reduction					0.121 (1)	0.728
	No	108 (64.3)	54 (65.1)	50 (62.5)		
	Yes	60 (35.7)	29 (34.9)	30 (37.5)		
Death					8.366 (1)	0.004
	No	94 (56.0)	56 (67.5)	36 (45.0)		
	Yes	74 (44.0)	27 (32.5)	44 (55.0)		

* Prior endocrine therapy categories were not mutually exclusive, as some patients received more than 1 endocrine agent sequentially in the metastatic setting before CDK4/6 inhibitor initiation. Therefore, the sum of treatment categories exceeds the number of individual patients. Categorical variables were compared using Pearson’s chi-square test or Fisher’s exact test, as appropriate. Pearson’s chi-square statistics (χ^2^) and degrees of freedom (df) are reported. For comparisons performed using Fisher’s exact test, only the *p*-value is presented. A *p*-value < 0.05 was considered statistically significant. Abbreviations: BMI, body mass index; ECOG, Eastern Cooperative Oncology Group; CDK4/6, cyclin-dependent kinase 4/6; MMRI, Metabolic–Muscle Reserve Index.

## Data Availability

The datasets generated and/or analyzed during the current study are available from the corresponding author upon reasonable request. The data are not publicly available due to patient privacy and confidentiality considerations.

## Supplementary Materials

The following supporting information can be downloaded at https://www.mdpi.com/article/10.3390/jcm15218641/s1. Supplementary Table S1: Assessment of the proportional hazards assumption using Schoenfeld residuals. Supplementary Table S2: Comparison of Prognostic Discrimination Between MMRI and Its Individual Components. Supplementary Table S3: Incremental Prognostic Performance and Bootstrap Internal Validation of MMRI.

## Abbreviations

The following abbreviations are used in this manuscript:

HR+	Hormone receptor-positive
HER2−	Human epidermal growth factor receptor 2-negative
CDK4/6	Cyclin-dependent kinase 4/6
MMRI	Metabolic–Muscle Reserve Index
PFS	Progression-free survival
OS	Overall survival
PET/CT	Positron emission tomography/computed tomography
SUVmax	Maximum standardized uptake value
LDH	Lactate dehydrogenase
PVI	Psoas volume index
ROC	Receiver operating characteristic
MBC	Metastatic breast cancer
NLR	Neutrophil-to-lymphocyte ratio
SMI	Skeletal muscle index
BMI	Body mass index
ECOG	Eastern Cooperative Oncology Group
CRP	C-reactive protein
CA 15-3	Cancer antigen 15-3
CT	Computed tomography
FDG	Fluorodeoxyglucose
SPSS	Statistical Package for the Social Sciences
