# Supplementary material for: Prognostic Value of Metabolic–Muscle Reserve Index in HR+/HER2− Metastatic Breast Cancer Treated with CDK4/6 Inhibitors
Source: J Clin Med. 2026 Nov 6;15(21):8641. doi: 10.3390/jcm15218641 (PMC13764139; doi:10.3390/jcm15218641)
Supplement: Supplementary file 1 [file jcm-15-08641-s001.zip › jcm-4651404-supplementary.pdf]

Supplementary Table S1. Assessment of the Proportional Hazards Assumption Using Schoenfeld Residuals

Variable	PFS			OS		
	χ^2	df	p-value	χ^2	df	p-value
ln-MMRI	2.0243	1	0.15	1.3411	1	0.247
Liver metastasis	1.8486	1	0.17	0.0115	1	0.915
Bone metastasis	—	—	—	0.8399	1	0.359
Lymph node metastasis	0.0400	1	0.84	0.5560	1	0.456
Ki-67	0.4165	2	0.81	—	—	—
Brain metastasis	0.9488	1	0.33	0.0282	1	0.867
Prior endocrine therapy	0.0347	1	0.85	1.2795	1	0.258
CDK4/6 treatment line	0.2242	1	0.64	3.2591	1	0.071
**Global test**	**7.6717**	**8**	**0.47**	**6.6009**	**7**	**0.472**

Abbreviations: PFS, progression-free survival; OS, overall survival; MMRI, Metabolic–Muscle Reserve Index; CDK4/6, cyclin-dependent kinase 4/6; df, degrees of freedom.

Supplementary Table S2. Comparison of Prognostic Discrimination Between MMRI and Its Individual Components

Variable	OS C-index (SE)	PFS C-index (SE)
MMRI	0.652 (0.038)	0.625 (0.033)
SUVmax	0.565 (0.039)	0.591 (0.034)
LDH	0.602 (0.041)	0.565 (0.033)
Albumin	0.617 (0.039)	0.548 (0.032)
PVI	0.535 (0.038)	0.536 (0.033)

Abbreviations: MMRI, Metabolic–Muscle Reserve Index; OS, overall survival; PFS, progression-free survival; SE, standard error; SUVmax, maximum standardized uptake value; LDH, lactate dehydrogenase; PVI, psoas volume index.

Supplementary Table S3. Incremental Prognostic Performance and Bootstrap Internal Validation of MMRI

**Parameter**	**PFS**	**OS**
Clinical model C-index	0.678	0.698
Clinical model + MMRI C-index	0.700	0.721
Optimism-corrected C-index, clinical model	0.649	0.681
Optimism-corrected C-index, clinical model + MMRI	0.671	0.704
Likelihood-ratio χ^2	12.254	13.115
Likelihood-ratio p-value	0.000464	0.000293

Abbreviations: MMRI, Metabolic–Muscle Reserve Index; PFS, progression-free survival; OS, overall survival.

The PFS clinical model included liver, lymph node, and brain metastases, Ki-67, prior endocrine therapy, and treatment line. The OS clinical model included liver, bone, lymph node, and brain metastases, prior endocrine therapy, and CDK4/6 treatment line. MMRI was included as a log-transformed continuous variable. Internal validation was performed using 1,000 bootstrap resamples to estimate optimism-corrected Harrell's C-indices. Likelihood-ratio p-values indicate improvements in model fit.
